# Systematic evaluation of integration between China’s digital economy and sports industry: Two-stage grey relational analysis and vector autoregressive model

**DOI:** 10.1371/journal.pone.0303572

**Published:** 2024-05-13

**Authors:** Xu Sun, Lide Su, Bo Zhou, Te Bu, Yang Zhang

**Affiliations:** 1 College of Physical Education, Hunan Normal University, Changsha, China; 2 School of Humanities, Inner Mongolia University of Technology, Hohhot, China; 3 HEHA CAT Sports Science and Technology Institute, Changsha, China; 4 Independent person, Windermere, FL, United States of America; Federal University of Goias: Universidade Federal de Goias, BRAZIL

## Abstract

**Objectives:**

The development of the digital economy constitutes a key component of China’s endeavors to advance towards “Digital China.” The sports industry functions as a new catalyst for high-quality economic growth. This study systematically evaluated the integration between these two sectors.

**Methods:**

First, we conducted two levels of grey relational analysis to assess their integration between 2016 and 2021. Second, we conducted a VAR analysis to determine whether their integration between 2009 and 2021 represents a causal relationship.

**Results:**

At the macro level, the grey relational analysis reveals that the sports industry (grade = 0.770) ranked second among China’s eight key economic sectors in terms of digital economy integration. At the meso level, a wide variation (ranging from 0.606 to 0.789) existed in the grade of integration between the digital economy and the sub-sectors of the sports industry. According to the VAR model, the digital economy does not Granger cause (p = 0.344) the growth of the sports industry.

**Conclusions:**

This study yielded two added values to the existing literature: First, there exists a sectoral imbalance in the digitization process; second, the explosive growth of the sports industry was not primarily caused by the digital economy. Accordingly, the “sports + digital” complex is still in the first wave of technological integration. We propose three policy recommendations, namely, sectoral synergistic development, overtaking via esports IP, and new economy and new regulation. Collectively, these findings provide updated insights for the digital transformation towards “building a leading sports nation” and “Digital China.”

## 1. Introduction

The world has seen a period of fewer than four centuries from its initiation of the Industrial Revolution. In the annals of planetary history, a span of 400 years is a fleeting moment. However, within the context of human civilization, the advent of the Industrial Revolution instigated a momentous socioeconomic metamorphosis. This epoch stands as an unparalleled and expeditious time of progress, yielding substantial benefits for humanity. In the year 2023, during the transition to the era of Industry 4.0, the advent of large language models provides humanity with an initial peek into the structural transformations that will arise from the ongoing digitization process in the future. The digital economy stands as one core manifestation of the next wave of the Industry 4.0 revolution.

Since the establishment of New China, China has learned experiences of other developed countries in industrial development and developed a Chinese path to modernization [1]. Currently, as we stand on the cusp of a new era in history, China is strategizing to establish a dual circulation strategy that aligns with its cultural values. China’s approach to this transformation is rooted in “Digital China” by leveraging the potential of the digital economy. In less than a decade, the digital economy has emerged as a significant catalyst for China’s sustained productivity [2]. In 2022, the report of the 20th National People’s Congress emphasizes “We will accelerate the development of the digital economy, further integrate it with the real economy, and build internationally competitive digital industry clusters.” This suggests that, within the evolving development landscape, China’s economy can achieve both qualitative and quantitative advancements by integrating the digital economy with the real economy. Hence, the evaluation of the level of integration between China’s traditional sectors and the booming digital economy holds major theoretical and practical significance.

It is important to first establish a clear definition of the digital economy. For this purpose, we can consult the definitions of the United States and China, both of which presently hold the top positions globally in terms of the gross output of their digital economies. According to the U.S. Department of Commerce [3], the digital economy includes “(1) the digital‐enabling infrastructure needed for a computer network to exist and operate, (2) the digital transactions that take place using that system (“e‐commerce”), and (3) the content that digital economy users create and access (“digital media”).” The National Bureau of Statistics of China’s definition of the digital economy encompasses a range of economic activities wherein data resources play a crucial role as factors of production [4]. It emphasizes the significance of modern information networks and underscores the key role of information and communication technologies in driving efficiency improvements and optimizing the economic structure. Both countries place significant importance on data resources as a fundamental component of production. At the same time, it is notable that each country exhibits a unique focus area: the United States places significant attention on the technological foundations that underpin the digital economy; by contrast, China assumes a leading role in the realm of retail e-commerce and digital payment applications. Hence, this study focuses on the economic aspect of development, specifically assessing the level of integration between industrial economic development and the digital economy.

The integration of the digital economy with retail sales has become the new norm within contemporary Chinese life, and signifies a forthcoming direction of industrial integration across all levels. Among the many economic sectors, the sports industry is undergoing comprehensive digital economic integration due to the multiple-level overlap between its industrial elements and digital technologies. For example, the increasing use of smartphones and mobile platforms has led to the recognition of sports-related exercise data as an important vertical for technological titans [5]. The popularization of sports media live streaming on mobile platforms has been facilitated by the expansion of digital infrastructure, leading to a progressive decline in market share for the traditional live broadcasting sector. The exponential growth of e-commerce has drastically changed consumer behavior, prompting sporting goods manufacturers to continuously monitor the prevalence of platform economy to refine their business models. The integration of the digital economy can lead to enhanced management efficiency within the sports industry, hence facilitating its overall growth [6]. Similarly, the expansion of the sports industry would in turn stimulate the advancement of the digital economy and lead to the emergence of a new “sports + digital” complex. The sports industry holds a unique position within China’s economic structure, serving as a vital component for both the real economy and public health [1]. Thus, the digital transformation of this sector carries immense importance.

Conducting objective analyses of the integration situation between China’s digital economy and the sports industry is crucial for understanding the interplay and inherent relationship between these two sectors. Furthermore, it serves as a pivotal aspect in formulating a comprehensive strategy for “building a sports power” and advancing the process of “Digital China,” as outlined in key government policies. In this regard, there is no shortage of research available in the Chinese context. In a theoretical review, Ren and Huang provided an in-depth explanation of how the digital economy may effectively facilitate the advancement of the sports industry’s high-quality development [7]. It is proposed that at the macro level, the digital economy has the potential to enhance the input of production factors, alter resource allocation processes, and enhance overall factor productivity. At the meso level, the digital economy can facilitate organizational changes within the sports industry, encourage the optimization and advancement of its structure, and foster cross-border integration. At the micro level, the digital economy can stimulate sports enterprises to achieve economies of scale, economies of scope, and the long-tail effect. In a recent study conducted by Shen and colleagues, a theoretical framework of integration was proposed to analyze the restrictions and change mechanisms within the sports industry during the process of digital transformation [8]. The authors put out the notion that industrial policy, consumer demand, and the digital market serve as external driving forces. On the other hand, the endogenous forces primarily include the competition driving force, technology change force, and platform support force. Considering the practical challenges, the authors proposed a four-tier implementation approach for establishing a digital sports consumption pattern. Other scholars have initiated a comprehensive theoretical discourse regarding the practical integration of digital technologies, such as big data [9], Internet+, and blockchain [10], within the sports industry.

The problem here is threefold. First, there is a dearth of empirical data about the integration of these two sectors. This deficiency is particularly pronounced when comparing the quantity of theoretical studies to quantitative analysis. The focal point of research on the digital transformation of the sports industry should revolve around devising a scientifically rigorous approach for assessing the extent of their integration. In this context, the current body of econometric research mostly centers around the coupling coordination model [11–13]. For example, Wang et al. [11] analyzed provincial-level data spanning the years 2015 to 2019 and their findings suggest that the degree of coupling coordination between China’s digital economy and sports industry was relatively low, with an imbalanced development pattern. In a recent study, Ren conducted an examination of the coupling coordination between these two sectors at the national level from 2014 to 2021 [12]. Ren developed an index system consisting of 19 indicators and it was determined that the degree of coupling coordination increased from 0.0539 to 0.7044, indicating a positive trend of coordinated development. Nevertheless, there is a disconnect between ample theoretical frameworks and few empirical analyses.

Second, the analysis of panel data indicates that the sports industry is positively influenced by the digital economy [14], but it is important to note that not all sub-sectors within the industry exhibit the same level of impact from this underlying trend. It is perhaps fair to assert that the online retail sector for sporting goods is rapidly adopting the e-commerce model, but it cannot be extrapolated that other sub-sectors are embracing a comparable rate of digitization. Our recent SWOT-AHP analysis shows that the sports education business, despite facing a critical juncture, is nowhere near employing digital platforms or new IT technologies to effectively enhance its market position [15]. To our knowledge, there is no empirical analysis of the integration within sub-sectors of the sports industry into the digital economy. An exhaustive analysis of the current circumstances is essential for formulating policy recommendations for an efficient “sports + digital” complex.

Third, a widely recognized mantra in the field of statistics asserts that “association is not causation.” In practice whether or not an association may be attributed as the cause has to be considered on top of various other factors. Such evaluation should not solely rely on the dynamics associated with different indicators, even if they appear to be highly predictive of two relevant things. The coupling coordination model can be characterized as a form of reciprocal coupling rather than causal coupling, for example. Recently, Huang and colleagues conducted a mediation analysis and described a three-way mediating path of a stronger digital economy, a stronger technology level, and a stronger sports industry [16]. The authors concluded that the digital economy has an indirect effect on the development of the sports industry by enhancing scientific innovation. Likewise, neither of the mediation effects provides conclusive evidence of a causal relationship.

To this end, our focus lies in examining the integration of sub-sectors within the sports industry into the digital economy. We are particularly interested in answering, whether the rapid expansion of the sports industry may be caused by the digital economy, and if so, whether there exists a bidirectional causal relationship between the two.

Here, we propose two comprehensive methods for addressing the aforementioned gaps. First, grey relational analysis is the extension of the grey system theory [17], whose fundamental concept is based on the geometry of the sequence curve to determine whether the connection between different sequences is close: the higher the correlation between the different sequences, the closer the characteristic index of grey relational grade is to unity, and vice versa. In addition, grey relational analysis is more adept at overcoming measurement challenges posed by small samples, partial information unknown, and poor information in real-world uncertainty problems [18]. Therefore, the selection of grey relational analysis to evaluate the integration of the sports industry into the digital economy is scientifically sound and practically feasible.

Second, the vector autoregression (VAR) model is a statistical model that considers each endogenous variable in the system as a function of its own lagged values as well as the lagged values of all other endogenous variables. This approach, especially in econometrics [19], helps to mitigate the potential bias associated with the traditional structured model, thereby enhancing the model’s ability to capture dynamics among variables. The Granger-causality test can be employed on the VAR model to examine the presence of a causal relationship between the growth of the digital economy and the expansion of the sports industry. The statistics of the VAR model are typically summarized using impulse response functions and forecast error variance decompositions. These analytical techniques can be employed to predict the inherent correlation and contribution of mutual support between these two sectors in shaping the characteristics of the “sports + digital” complex.

In the latest 14th Five-Year Plan for Sports Development (2021–2025) [20], the government states that “Digital sports involves the practical application of digital technology to enhance the advancement of sports. The scope primarily encompasses the implementation of digital governance in the sports sector, the digitization of national fitness initiatives, the digital transformation of sports training, the digitalization of sports competitions, and the digitalization of the sports industry.” Within the same document, the digital sports construction project is positioned as the 22nd priority focus and has six significant tangible goals: Establishing a unified network for national fitness services; expediting the digitalization of sports venues and facilities; developing a sports event information management platform; establishing multiple digital sports laboratories; constructing a sports data framework comprising the National Sports Data Center and regional sports data centers; and enhancing the infrastructure for sports informatization. Accordingly, conducting a thorough assessment of the present state of the digitization process in the sports industry and its sub-sectors holds major policy relevance. Therefore, this study uses grey relational analysis and the VAR model to analyze the integration between China’s digital economy and the sports industry. The purpose of this study is to present analytical recommendations for strengthening the development of the digital sports industry. Ultimately, the findings are expected to provide theoretical knowledge and practical recommendations that can help achieve the long-range goals of “building a leading sports nation” and “Digital China.”

## 2. Methods

### 2.1 Grey relational analysis

The SPSSPRO was used to conduct a grey relational analysis of data at both the macro and meso levels. In step 1 of grey relational analysis, the characteristic sequence is defined as *Y*_0_, and factor sequences are defined as *X*_i_(*t*), where *i* denotes the number of relevant factors and *t* denotes the time series. In step 2, the original data are standardized to dimensionless values using the initialization method. The initialization method is given by Eq (1):

xi'(k)=xi(k)x(1)(x(1)≠0) (1)

In step 3, the absolute difference sequence is calculated as Eq (2):

$$\Delta_{i}(t)=|Y_{0}(t)-X_{i}(t)|$$ (2)

In step 4, the grey relational coefficient is calculated as Eq (3):

$$R_{i}(t)=\frac{\underset{i}{\min}\;\underset{t}{\min}|Y_{0}(t)-X_{i}(t)|+\rho \cdot \underset{i}{\max}\;\underset{t}{\max}|Y_{0}(t)-X_{i}(t)|}{\left|Y_{0}(t)-X_{i}(t)|+\rho \cdot \underset{i}{\max}\;\underset{t}{\max}|Y_{0}(t)-X_{i}(t)\right|}$$ (3)

where *ρ* is the resolution ratio, which is generally taken as 0.5, as in this study.

In step 5, finally, the grey relational grade is calculated as Eq (4):

$$r_{i}=\frac{1}{n}\sum_{t=1}^{n}R_{i}(t)$$ (4)

Here, the higher the grade value, the stronger the integration between the characteristic sequence and factor sequences.

### 2.2 Vector autoregressive model

The Eviews 13 was used to model the added value of the digital economy and the total scale of the sports industry and constructed a recursive VAR model to further analyze the causal relationship between the sports industry and the digital economy. The VAR model is expressed as Eq (5):

$$Y_{t}=A_{1}Y_{t‐1}+A_{2}Y_{t‐2}+\ldots +A_{k}Y_{t-k}+\varepsilon_{t},(t=1,2,\ldots ,k)$$ (5)

where, *t* denotes the time series, *Y*_t_ denotes the *n*-dimensional endogenous variables, A1, A2,…, Ap denotes a *n* × *n* coefficient matrix, ɛ_t_ denotes the random disturbance term, and *k* denotes the lag order corresponding to the variable.

For brevity and clarity, this study presents detailed computations within the Eviews 13 alongside the corresponding results. The resultant VAR model was used to delve deeper into the level of advancement in the “sports + digital” complex.

### 2.3 Data range

The complete statistics (S1–S3 Tables) that support the conclusions of this study are available on figshare (DOI: 10.6084/m9.figshare.24053820.v1). In 2014, the State Council issued Document No. 46 [21] to steer the development trajectory of China’s sports industry, which decisively determined the significance of the sports industry in the overall socioeconomic development. Before this, there was a lack of properly established comprehensive statistical data for the sub-sectors of the sports industry. One year later, in response to this new development, the National Bureau of Statistics of China revised the National Statistical Classification of the Sports Industry [22], which provides a much more detailed picture of the sports industry as reflected in its annual bulletins. For the grey relational analysis, we therefore collect government statistics (see also S1 and S2 Tables) from the year 2016. To further assess the quality of market development, we utilize the added value data to construct an index system for the digital economy and sports industry (see the following section for more details). When developing the VAR model index system, it is crucial to consider the temporal continuity of the time series and ensure that the dataset has a sufficient number of samples. Thus, the data range is expanded to include the year 2009 (see also S3 Table), which represents the earliest accessible data pertaining to China’s digital economy development. Nevertheless, because of the absence of public data on the added value of the national sports industry before 2015, we made the tactical choice to use the total scale statistics as a proxy indicator for the sports industry in the VAR model.

Accordingly, the statistics of the digital economy are extracted from the Blue book of digital economy: Analysis and forecast of China’s digital economic situation (2021) [23]; the statistics of the sports industry are extracted from the statistical bulletins published by the National Bureau of Statistics of China and the General Administration of Sport of China; the statistics of other major economic sectors are extracted from the China Statistical Yearbook.

### 2.4 Index system

The macro-level index system applied in the grey relational analysis examines the extent to which China’s digital economy is integrated with the sports industry and other sectors between 2016 and 2021. In selecting specific indicators, the characteristic sequence (Y) is represented by the added value of China’s digital economy. The factor sequence (X_1_) is represented by the added value of the sports industry, while additional factors such as the total scale of culture and related industries (X_2_), the added value of tourism and related industries (X_3_), the added value of the industrial sector (X_4_), the added value of construction enterprises (X_5_), the added value of wholesale and retail trades (X_6_), the added value of hotels and catering services (X_7_), and the added value of financial intermediation (X_8_) are also introduced. Among these sequences of factors, X_4_ and X_5_ are classified within the secondary industry, while the remaining factors are classified within the tertiary industry.

The meso-level index system applied in the grey relational analysis examines the extent to which China’s digital economy is integrated with the sub-sectors of the sports industry between 2016 and 2021. The characteristic sequence (Y’) is represented by the added value of China’s digital economy. The value added of each sub-sector within the sports industry is established as a factor series, including sports management activities (X_1_’), sports competition performance activities (X_2_’), physical fitness and leisure activities (X_3_’), management of sports venues and facilities (X_4_’), sports brokerage and agency, advertising and exhibition, performance and design services (X_5_’), physical education and training (X_6_’), sports media and information service (X_7_’), sales, leasing and trade agency of sporting goods and related products (X_8_’), other sports services (X_9_’), manufacture of sporting goods and related products (X_10_’), and construction of sports facilities (X_11_’).

**Fig 1:**
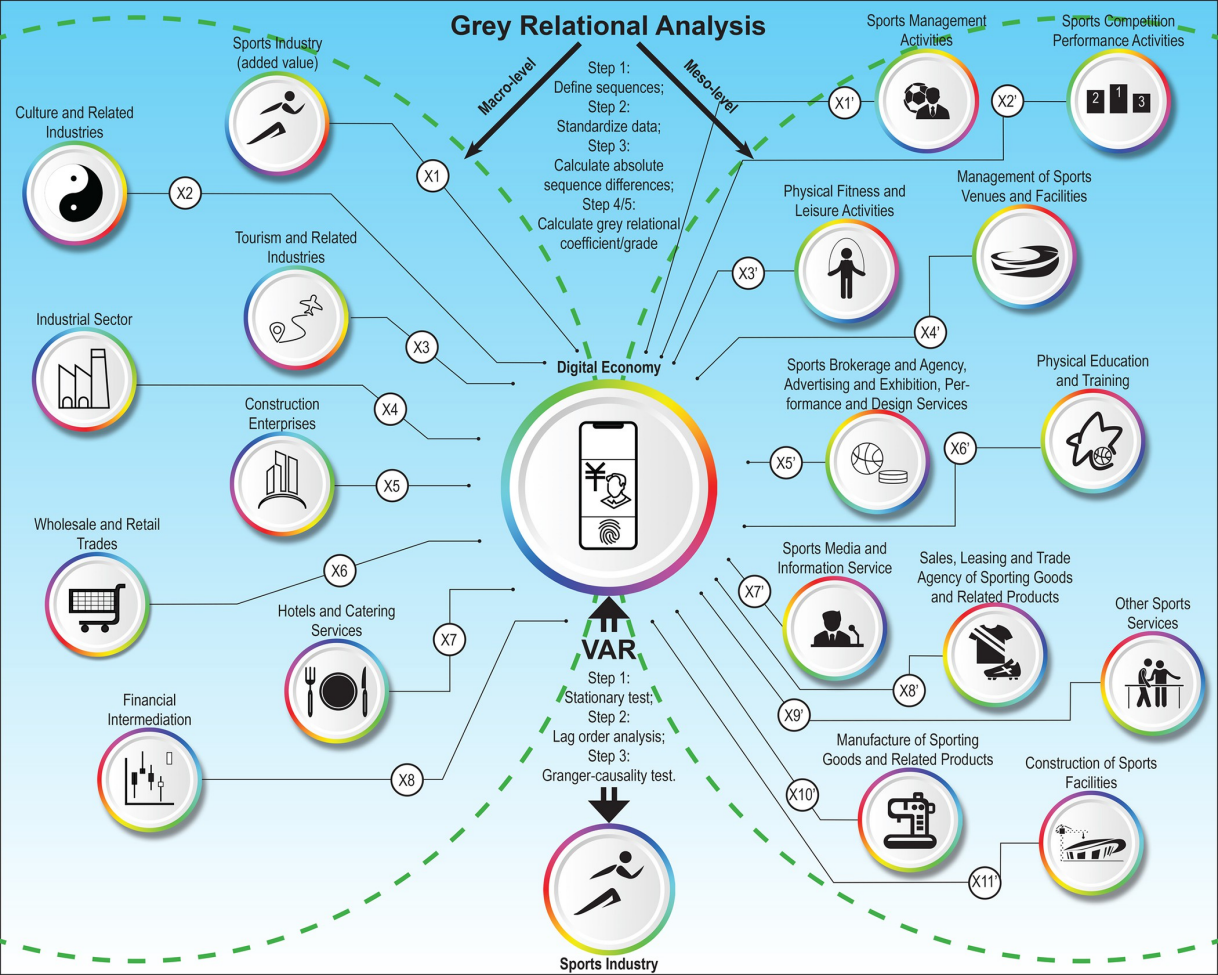
Two-stage grey relational analysis and vector autoregressive (VAR) model.

For the VAR model, the index system consists of the added value of China’s digital economy (X”) and the total scale of the sports industry (Y”) between 2009 and 2021. Fig 1 overviews the index system.

## 3. Empirical evidence from the grey relational analysis

### 3.1 Macro-level integration

Table 1 presents an overview of the grey relational grades about the relationship between China’s digital economy and eight main economic sectors, covering the period from 2016 to 2021. A grey relational grade exceeding 0.5 indicates the presence of a specific relationship, with values closer to 1 indicating a stronger association between the measured elements, or a greater level of integration, as observed in this study. In general, the grey relational grade between China’s digital economy and main economic sectors exceeds 0.6, suggesting the emergence of an overall integration tendency throughout the period under study. The order of the integration of the digital economy, as determined by the grade of each economic sector, is as follows: X_2_ > X_1_ > X_5_ > X_8_ > X_7_ > X_3_ > X_4_ > X_6_.

**Table 1:** The grey relational grades of China’s digital economy with the sports industry and other major economic sectors.

Indicator	X_2_	X_1_	X_5_	X_8_	X_7_	X_3_	X_4_	X_6_
**Grade**	0.802	0.770	0.734	0.727	0.725	0.686	0.662	0.650
**Ranking**	1	2	3	4	5	6	7	8

To our knowledge, this study represents the first attempt to apply grey relational analysis to examine the integration of the digital economy and key economic sectors in China. The highest integration is observed between the digital economy and the culture and related industries (X_2_), with a value of 0.802. Following closely is the integration with the sports industry (X_1_), which reaches a grey relational grade of 0.770, ranking second. The emergence of these two sectors’ leading positions should not come as a surprise. In the context of China, the cultural industry and related industries have undergone a digital transformation since the early 1990s, exemplified by the shift from music tapes to digital music videos, in response to the global adoption of digital media [24]. Meanwhile, the process of the digital cultural industry is facilitating the swift integration of the digital economy and the sports industry [25]. The FIFA World Cup 2022 witnessed a milestone in China’s digital sports industry, as the cumulative direct on-demand views from TV and mobile devices surpassed 2 billion. This remarkable success has expanded the scope of sports-related consumption to unprecedented levels, such as the online sales of China sports lottery in 2022. Furthermore, the advent of digital media times gives rise to a novel form of attention economy and propels the growth of the digital sports industry [25]. Around the world, individuals who utilize X (formerly known as Twitter), Instagram, and other social media platforms, actively express their opinions on live sports broadcasts, share their emotions, and engage in a novel form of interactive sports “participation” experience [26, 27]. In China, the emergence of Douyin (the Chinese equivalent of TikTok) and Bilibili has empowered the younger generation to create pop-culture memes and distribute e-commerce advertisements, resulting in a profound transformation in the way sports are consumed [28, 29]. The interconnection of the digital economy, cultural and related industries, and the sports industry are intricately intertwined, as advancements in any of these areas mutually enhance their synergistic development.

From another angle, the digital economy further bolsters the resilience of the sports industry when confronted with challenges. Amidst the COVID-19 epidemic, the sports industry experienced a decline of nearly 10% in added value from 2019 to 2020 (see also S1 Table); however, this sector demonstrated a swift recovery and attained new industrial records in 2021. In particular, demands for online sports consumption such as home-based streaming fitness and esports viewing have surged ever since [30, 31] and have now become the new norm within the digital sports industry. Such economic resilience during this unprecedented period can be attributed to high-level digital access, which has also been observed in other economies despite different socioeconomic structures [32]. In Spain, the digitization of sports services has played a critical role in managing the crisis and facilitating the recovery of small and medium-sized enterprises [33]. Together, the relatively high integration between the digital economy and the sports industry reinforces our notion that the future success of the Chinese and global sports industry depends on the rate and level at which it adopts digital technologies.

It is noteworthy that the integration among construction enterprises (X_5_), financial intermediation (X_8_), and hotels and catering services (X_7_) sectors and the digital economy exceeded 0.7, suggesting the increasing share of digital transformation within socioeconomic development. Collectively, this macro-level grey relational analysis provides an overview of the integration between China’s digital economy and the sports industry, along with other main economic sectors for comparative purposes.

### 3.2 Meso-level integration

The meso-level grey relational analysis focuses on sub-sectors within the sports industry and their integration with the digital economy. The objective is to gain in-depth knowledge, specifically regarding the sectoral strengths as well as weaknesses, beneath the overall process of digital transformation. As shown in Table 2, the order of integration between the sub-sectors of the sports industry and the digital economy is as follows: X_4_’ > X_1_’ > X_2_’ > X_8_’ > X_11_’ > X_6_’ > X_9_’ > X_7_’ > X_3_’ > X_10_’ > X_5_’. This analysis thus identifies the presence of a sectoral imbalance in the digitization process.

**Table 2:** The grey relational grades of China’s digital economy with sub-sectors of the sports industry.

Indicator	X_4_’	X_1_’	X_2_’	X_8_’	X_11_’	X_6_’	X_9_’	X_7_’	X_3_’	X_10_’	X_5_’
**Grade**	0.789	0.785	0.778	0.745	0.724	0.706	0.670	0.641	0.638	0.632	0.606
**Ranking**	1	2	3	4	5	6	7	8	9	10	11

The three highest-ranked sectors are management of sports venues and facilities (X_4_’), sports management activities (X_1_’), and sports competition performance activities (X_2_’). These rankings reflect the fact that China has been hosting a significant number of prestigious sporting events in recent years. Since the 2000s, China has successfully organized two Olympic Games and various mega events within a condensed two-decade frame. High-specification sporting events are inseparable from high-quality sports management, which are complementary and necessitate substantial investment in the digitization of sporting facilities [34] and the organization of sporting events [35]. As an illustration, the main venue of the 14th National Games of China, serving as China’s first stadium with full 5G connectivity, is equipped with over 2,000 Wi-Fi 6 networks [36]. Moreover, China is at the forefront of the movement to establish esports as a recognized and governed version of sports [37]. China through its IT titans has hosted several prestigious esports championships [38], acquiring valuable expertise in organizing such events. This significantly advances China’s sports management in the realm of esports. As a result, China has been able to amass considerable competence in the domain of digitized sports management.

Furthermore, three additional sub-sectors appear to be well integrated with the digital economy. First, sales, leasing and trade agency of sporting goods and related products (X_8_’) has experienced significant growth due to the widespread adoption of e-commerce. A recent study demonstrates the potential of digital innovation to boost the export of sporting products. Zhao and colleagues analyzed the panel data of 28 Chinese provinces spanning from 2013 to 2020 [39]. Their analysis indicates that technological innovation efficiency acts as an intermediary factor between the digital economy and the export competitiveness of sporting goods. It is no surprise that such technological advancement has prompted a swift transition from the traditional sales approach of utilizing franchised distributors to the utilization of e-commerce platforms, the live-streaming sector, and the WeChat business landscape among Chinese sports enterprises. Using ANTA Sports as an example, the sports apparel transactions on the Tmall at the “Double Eleven” event in 2021 exceeded a total of 3.389 billion yuan [40]. The COVID-19 pandemic further accelerated the digital transformation of this sector [41], including the adoption of the popular internet sales model in China known as time-limited live commerce [29]. Hence, it is evident that the utilization of digital technology has greatly simplified the tradings of sporting goods.

Second, although the sports industry as a whole is typically linked to low-carbon footprints, conventional sports facilities consume a significant amount of energy [42], prompting contemporary mega events organizers to prioritize constructing environmentally friendly sports facilities and implementing sustainable maintenance practices. Since China acceded to the WTO, there has been a surge in the hosting of international sporting events, which has led to the need for the development of new sports venues. Additionally, the implementation of the National Fitness Plan has accelerated the demand for public sports facilities [1]. Throughout this process, the Chinese government has integrated a range of digital technologies to address the issue of carbon emissions, environmental protection, and meet the increasing demands for sports facilities [43]. Other newly constructed stadiums and associated facilities, which have been financed by private entities [44], also have state-of-the-art digital infrastructure to fulfill the future demands of mega events. Moreover, China has been utilizing its vast expertise to assist in the construction and upgrading of sports venues and digital infrastructures in foreign countries in recent years [45]. These efforts have led to synergistic green development between sports facilities and urban upgrades [46], and as a result, we find that the construction of sports facilities (X_11_’) integrates well with the digital economy.

Third, the physical education and training (X_6_’) sector is catching up with the digitization process and leveraging new digital technologies to advance its growth. The success of Team China in the Olympic Games can be partially ascribed to their adoption of new technologies [47]. For example, Chinese athletes won RS:X windsurfing medals for the first time at the Tokyo 2020 Summer Olympics. This accomplishment was made possible through the utilization of advanced computational wind tunnel simulations, which involved millions of full-data simulations for skill optimization [48]. Digital technologies not only contribute to the success of Chinese athletes in international competitions, but they are also utilized in recreational fitness activities. These technologies have boosted the value of sports technology outputs, such as smart sports facilities, catering to everyday sports participants [49]. Furthermore, the “Double Reduction” policy fundamentally changes the future landscape of the Chinese education market [15], necessitating the adoption of brain science-associated technologies. Health-tracking technologies and data mining [50] can offer data-driven content optimization for children and adolescents in their developmental stages. Going forward, the physical education sector is compelled to fully incorporate itself into the digital economy to capture the market demands.

It is crucial to look into the shortcomings that have been identified through the grey relational analysis. It appears that other sports services (X_9_’) are experiencing a delay in their integration with the digital economy. According to the National Statistical Classification of the Sports Industry [22], other sports services include sports tourism activities, sports health services, sports lottery services, sports exhibition services, sports finance and asset management services, and sports technology and intellectual property services. Although the sector’s current contribution to the overall sports industry is rather modest, it plays a crucial role in expanding sports consumption. The snow and ice sports, for example, have historically had a dual purpose as both sports and tourism activities. However, its position was relatively insignificant until the Winter Olympics was hosted in China. Thus, the lack of integration between this business and the digital economy can be addressed by the implementation of supportive policies [51]. Developing sports health services is essential for implementing the “sports + preventive medicine” concept. This process can be expedited by reviewing international practices such as remote patient monitoring [52]. In a general sense, this sector possesses ample potential for progress by integrating additional digital technologies and embracing the digital economy [53].

The current state of the sports media and information service (X_7_’) sector indicates an important sectoral weakness. A Polish study reveals that younger cohorts exhibit a higher propensity to acquire sports-related information through social media platforms, whilst displaying a diminished inclination to watch live sports events on television [54]. Likewise, another survey about the Italian sports industry had similar findings, indicating that the use of digital channels is exponentially increasing, particularly among young people [55]. Here, the integration of the digital economy has limited relevance to this economically vital sector, highlighting the difficulties encountered by China’s sports media business, which is characterized by a small market share and a lack of market specialization. In the United States, this sector is characterized by competition among multiple market players [56], such as ESPN, HBO, Fox Sports, and Disney+. This competitive landscape facilitates ongoing market expansion and the provision of high-quality digital services to North American sports consumers. On the contrary, a limitation in the broad digital transformation of China’s media broadcasting industry is the lack of competition driven by market dynamics. The sociopolitical foundation in China places a strong emphasis on the predominance of state-owned enterprises within crucial economic sectors. Within the sports media sector, China Central Television, a traditional television-oriented media platform, assumes a prominent role in driving sectoral growth. However, it also imposes limitations on the expansion of private market players in this domain, which has been a known issue without much progress till today [57].

The limited integration of the physical fitness and leisure activities (X_3_’) sector, as well as the manufacture of sporting goods and related products (X_10_’) sector, with the digital economy presents enormous prospects. Physical fitness and leisure activities encompass a wide range of mass sports activities, serving as the fundamental pillar of Chinese sports and contributing to China’s future competitiveness [1]. The prevalence of daily digital services and commercial activities among Chinese citizens indicates a decoupled pace between this sector and the accelerated digitization trend in people’s lives. Consequently, an enormous amount of data generated in the realm of daily recreational exercise holds little value. However, this issue is not exclusive to China. In Italy, it has been found that there is a limited adoption of digital innovation technology (such as mobile apps and video tutorials on social media platforms) in the physical fitness sector (gyms/fitness centers) [55]. Thus, the current state can catalyze expediting the integration of digital technologies within the public sports service system. In the sector of manufacture of sporting goods and related products, which constitutes the largest component of the national sports industry, it is evident that Industry 4.0 has not yet made a substantial impact here. Our result aligns with the findings reported in previous research. Pan and Fang analyzed the input-output data of China in 2012, 2018, and 2020 to empirically assess the extent of integration development between the sports industry and the digital economy industry [58]. Their analysis determined that the adoption of digital technology applications in the sports manufacturing industry was restricted. The sports manufacturing sector is historically labor intensive. Nevertheless, as the industry gradually transits towards Industry 4.0, there is potential for improved output efficiency and energy conservation. These factors are crucial for establishing a competitive edge in the green economy.

Finally, although the integration of the sports brokerage and agency, advertising and exhibition, performance and design services (X_5_’) sector is rather low, this outcome may not be an unwelcome development. This sector is characterized as a small yet necessary component of the sports industry, and supports specialized employment. The integration of digital technology may not yield substantial gains in productivity, but it could increase unemployment rates. In our opinion, the sector is performing properly.

## 4. Empirical evidence from the VAR model

### 4.1 Stationary test

Econometric models necessitate the assumption of stationarity in economic time series. Before constructing the VAR model for analysis, it is necessary to conduct a stationary test. Initially, the raw data are transformed using natural logarithms to eliminate the impact of heteroskedasticity and temporal variations within the time series. Subsequently, we choose to conduct two types of stationary tests on the dataset. For the Dickey-Fuller GLS test, the null hypothesis is that the tested sequence presents a unit root. The lag length is based on the default Schwarz’s Bayesian criterion. Considering that not all sequences of economic variables have a constant and trend term, as assumed by the Dickey-Fuller GLS test, we proceed to calculate the non-unit root-based Kwiatkowski-Phillips-Schmidt-Shin test. This test is often preferred due to its excellent power and simplicity in various scenarios [59], as well as its ability to account for the limited sample size in this study. The underlying hypothesis posits that the sequence being evaluated exhibits either level or trend stationarity.

Table 3 shows the unit root and non-unit root-based stationary tests. With GLS demeaning, both the lnX’’ and lnY’’ reject, at the 5% critical value and significance level, the null hypothesis of the Dickey-Fuller GLS test following the application of a first-order differencing. The GLS detrending method reveals that the statistic of lnX’’ falls below the critical values, indicating that it is not stationary. On the other hand, the statistic of lnY’’ rejects the null hypothesis at the 1% critical value and significance level, indicating that it is stationary. In addition, the lnX’’ and lnY’’ are both found to be below the critical values of the Kwiatkowski-Phillips-Schmidt-Shin test at the 1% and 5% levels. As a result, we accept the null hypothesis of stationarity at the specified level. Despite some inconsistencies in the test findings, there is reasonable evidence to support the conclusion that the level series is a stationary sequence. Hence, it can be inferred that a long-term equilibrium relationship exists between the time series data of the digital economy and the sports industry.

**Table 3:** Stationary tests in first-order differencing.

Variable	Dickey-Fuller GLS					Kwiatkowski-Phillips-Schmidt-Shin			
	statiystic^a^	statistic^b^	1% CV*	5% CV*	10% CV*	statistic^a^	1% CV	5% CV	10% CV
**lnX”**	-2.722	-2.613	-2.792^a^	-1.978^a^	-1.602^a^	0.379	0.739	0.463	0.347
**lnY”**	-3.213	-4.598	-3.770^b^	-3.190^b^	-2.890^b^	0.301			

Note. CV, critical value.

^a^, constant only

^b^, constant and trend

*, test critical values are calculated based on 50 observations and may not be accurate for a sample size of 11 in this study.

### 4.2 Lag order analysis

The concept of degrees of freedom holds an essential place in VAR modeling [60]. In general, an increase in the number of variables and longer lag periods in the VAR model results in a decrease in the degrees of freedom in the estimating equations. Consequently, this loss in degrees of freedom may have an adverse effect on the accuracy of parameter estimation and result in false-positive findings of Granger-causality [61]. To provide a robust explanatory power of the parameters in the VAR model, it is necessary to strike a balance between the lag length and the degrees of freedom. Table 4 summarizes the lag structure. Based on the results obtained from the sequential modified LR test, Final prediction error, and Schwarz’s Bayesian criterion, it can be concluded that a lag of one is assumed to be the optimal lag at the 5% significance level [62].

**Table 4:** Lag-length criteria.

Lag	LogL	LR	FPE	AIC	SBC	HQ
**0**	5.883711	NA	0.001579	-0.776742	-0.716225	-0.843129
**1**	40.07401	47.86642*	3.91e-06*	-6.814802	-6.633251*	-7.013963
**2**	42.94229	2.868283	5.75e-06	-6.588458	-6.285873	-6.920393
**3**	48.38725	3.266974	6.90e-06	-6.877450*	-6.453831	-7.342159*

Note. LR, sequential modified LR test; FPE, Final prediction error; AIC, Akaike information criterion; SBC, Schwarz’s Bayesian criterion; HQ, Hannan-Quinn information criterion.

* Indicates lag order selected by the criterion.

### 4.3 Model specification

After verifying that the variables exhibit level stationarity, we proceed to use an automatic method to obtain the estimated VAR model using the optimal lag order. The model specification is presented in Table 5, and it is worth noting that the equation has a high *R*^2^ value.

**Table 5:** VAR model estimates.

Lag one	lnX”	lnY”
**lnX”(-1)**	1.004444	0.656611
	(0.06922)	(0.49754)
	[14.5106]	[1.31972]
**lnY”(-1)**	-0.035134	0.520296
	(0.03713)	(0.26685)
	[-0.94635]	[1.94979]
**constant**	0.411593	-2.843508
	(0.47023)	(3.37984)
	[0.87530]	[-0.84131]
***R*** ^**2**^_**adj.**_	0.995570	0.926332

Note. Standard errors in () and *t*-statistics in [].

### 4.4 Model diagnostics

**Fig 2:**
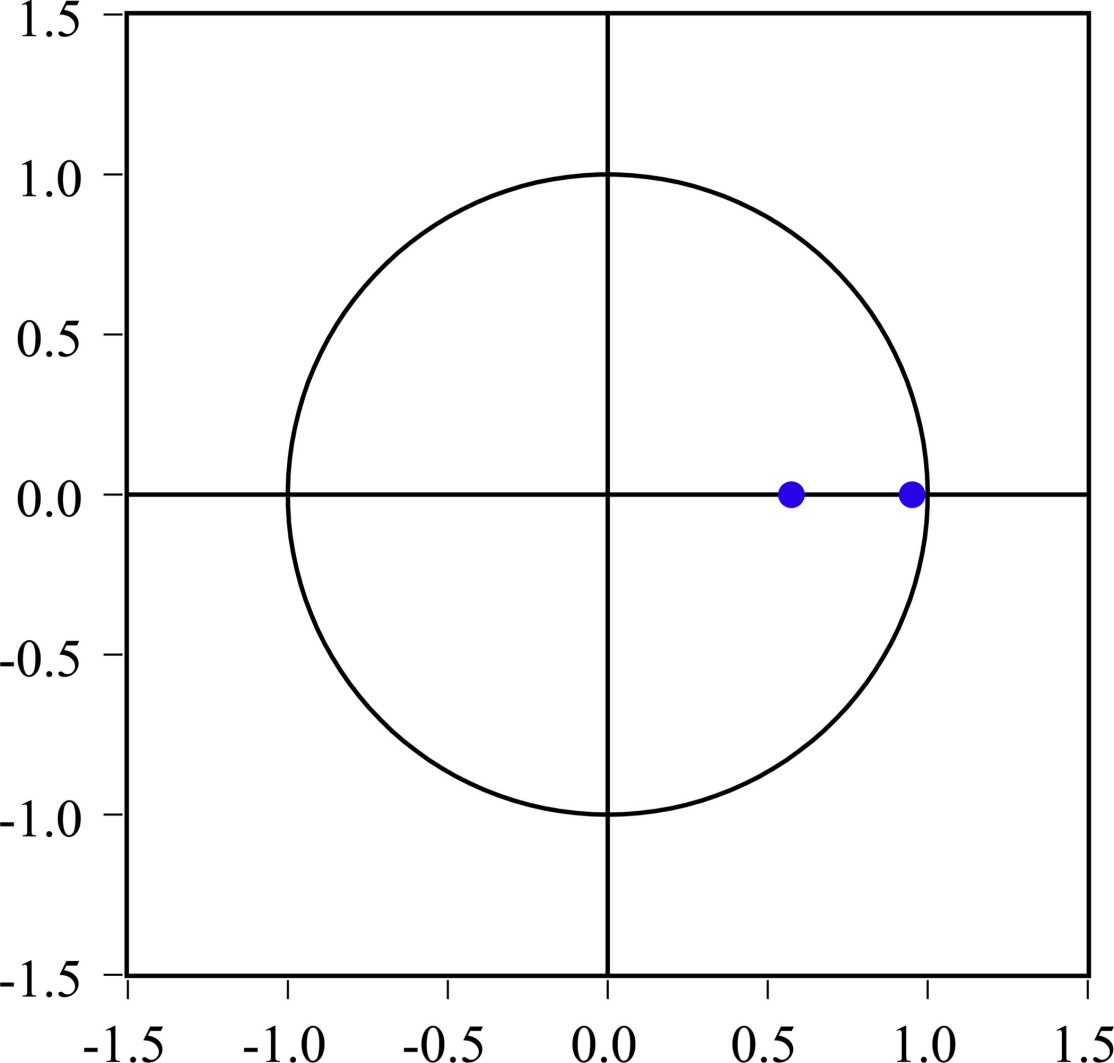
VAR roots of characteristic polynomial. Blue dots denote characteristic roots.

To assure the validity of subsequent analyses carried out with this VAR model, it is necessary to assess the stationarity condition and undertake residual analysis. The VAR roots depicted in Fig 2 exhibit the property that all inverse roots of the characteristic autoregressive polynomial possess a modulus that is smaller than one and is located within the unit circle. The lagrange multiplier test presented in Table 6 reveals that there is no significant serial correlation within lags 1 to 3. Therefore, it can be concluded that the VAR model exhibits stability and has no autocorrelation at the lag chosen.

**Table 6:** VAR residual serial correlation LM tests at lag n.

Lag	LRE* stat	df	Prob.	Rao F-stat	df	Prob.
**1**	1.715067	4	0.7880	0.423079	(4, 12.0)	0.7892
**2**	0.347796	4	0.9865	0.081344	(4, 12.0)	0.9866
**3**	1.914617	4	0.7515	0.476029	(4, 12.0)	0.7528

### 4.5 Impulse response functions

**Fig 3:**
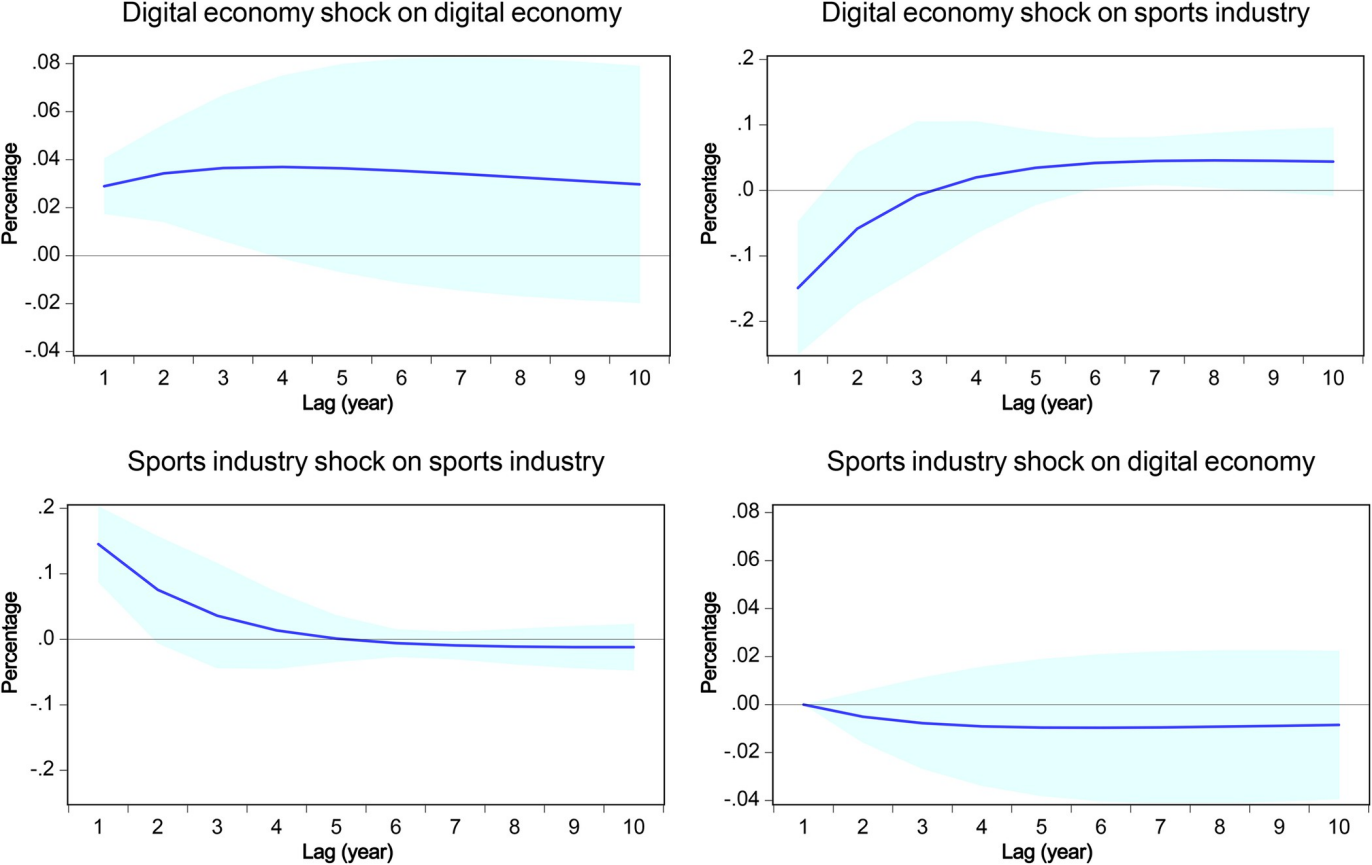
Impulse responses in the [digital economy]-[sports industry] recursive VAR model. The shaded regions depict the 95% confidence interval, which is calculated using analytic asymptotic standard errors.

Impulse responses trace out the response of the time path of each of the variables to a one-unit increment in the current value of one of the VAR errors, assuming that this error reverts to zero in subsequent periods while all other errors remain at zero. Fig 3 displays the impulse responses generated through applying the use of the degrees of freedom adjusted Cholesky decomposition method.

The upper panel, one main interest of this study, illustrates the impact of a one standard deviation increase in the digital economy on the variables. The potential for sustained growth in the added value of the digital economy may extend for a 10-year horizon. The initial shock has a detrimental effect on the total scale of the sports industry for approximately three years, followed by a sustained positive effect in the long run. These outcomes match with existing economic theory and empirical evidence. The growth of the digital economy can typically be attributed to technological innovations [63]. Such innovations contribute to the enhancement of the economic framework by facilitating streamlined transactions in digital services and goods [64], introducing new industrial structures for emerging applications [65], and promoting the widespread adoption of the Internet of Things [66]. As a result, these developments support continuous gains in the productivity of this sector. However, the transition towards superior efficiency in the digital economy has both intermediate-term and asymmetric impacts on other economic sectors. To illustrate this notion, the advent of ride-hailing operations enabled by smartphones has led to the decline of conventional taxi services and has ushered in a new era of the gig economy. However, the gig economy may face disruption as a result of the introduction of self-driving technology and a subsequent turbo-charged digital economy.

When it comes to the sports industry, the integration into the digital economy has mostly resulted in two levels of influence. The implementation of advanced digital technology necessitates financial investment and has an immediate impact on the industry’s gross margin. Chinese sporting goods enterprises have allocated resources towards R&D efforts [67]. These innovative solutions [68] are subsequently enhanced through efficient manufacturing processes and brand recognition. In recent years, Chinese sporting goods enterprises have successfully implemented technology-driven, cultural-integrated, and online-optimized brand management strategies to achieve superior performance in the domestic market [69], surpassing their foreign counterparts. This observation aligns with existing empirical evidence that maintaining a stable level of R&D investment yields positive influences on industrial growth [70], particularly in the context of China’s transition from low- to high-end positions within the global value chain [71]. Similarly, the digital economy in China is seeing rapid deployment and is leading the expansion of various economic sectors. This is primarily attributed to the widespread use of mobile payment systems, the emergence of e-commerce platforms, and the increasing popularity of live-streaming transactions. To keep pace with the advancements in the digital economy, the sports industry must enhance its infrastructure and corporate management strategy first. Consequently, it is not unexpected that this progression has a lagging impact on the industrial output. Upon the completion of this shift, the sports industry will experience a heightened rate of growth owing to the increased efficiency it brings. Using Li-Ning as a case point, it is observed that in the year 2021, the offline distribution channel experienced a growth rate in the high twenties, whilst the e-commerce sector witnessed a growth rate in the low forties [72]. Collectively, our analysis demonstrates a small, positive function of China’s digital economy in facilitating the development of the sports industry, ultimately yielding positive outcomes in the medium term.

The lower panel illustrates the impact of a one standard deviation increase in the sports industry on the variables. An unexpected rise in the total scale of the sports industry fades away over a span of four years and is associated with a marginal fall of ~0.01% in the added value of the digital economy. In the context of this study, it is useful to break down three distinct determinants that contribute to industrial output. One channel pertains exclusively to phenomena caused by events, such as the COVID-19 pandemic or the hosting of mega events. For instance, empirical evidence from China and other countries shows that hosting the Olympic Games has a sustained economic impact on the host nation [73], whereas other sporting events, such as ATP Masters 1000 Shanghai, Beijing 2015 IAAF World Championships, Dota 2 esports tournament, only result in impulsive effects on the local economy [74]. This phenomenon may serve as an analog for the analyses made in this study. The remaining two channels, which pertain to major government policy and fundamental industry upgrades, should not be classified as shock events and thus have no role in the impulse reaction functions.

Nevertheless, our data include a period that featured a groundbreaking policy implementation throughout China’s sports industry [21]. To demonstrate this impact, we proceed to compute the annual average growth rate (see also S3 Table). Over the course of the 13 years under examination, the digital economy had a remarkable growth rate of 13.23%, whilst the total scale of the sports industry even reached a 24.31% growth rate. To clarify, the sports industry demonstrates a much higher absolute rate of growth compared to the digital economy, which is not adequately captured in the impulse reaction functions, hence readers should not over-interpret this particular finding. In other words, the presence of a qualitative information variable, which can not be taken into account in the VAR model, is of significant importance in this context. This aspect will be addressed in further detail in later sections.

### 4.6 Variance decompositions

Table 7 summarizes the variance decompositions in the recursive VAR model, which assesses the percentage of the error made forecasting a variable over time due to a specific shock. The use of a 10-year horizon is intended to capture both short-term and long-term effects. For the digital economy, shocks of the sports industry explain merely 2.52% of the error variance in the forecast at the near horizon and this contribution slightly rises to 5.66% in the long horizon. This finding has two practical ramifications. On the one hand, the sports industry holds a limited position within the broader digital economy and it does not possess any predictive influence in the single variable VAR model being considered. On the other hand, the digital economy has consistently exhibited a contribution rate exceeding 90%, underscoring the predominant role of information technology in facilitating its rapid expansion. For the sports industry, shocks of the digital economy account for approximately 50% of the unexplained variability over a 10-year horizon. As explained in the preceding and next sections, the present VAR model possesses certain limitations stemming from its inability to quantify variables of a qualitative nature. To overcome this limitation, future research endeavors can be directed toward employing higher-level statistical methods.

**Table 7:** Variance decompositions from the VAR model ordered as the digital economy and sports industry.

Horizon	Variance decomposition of lnX”		Variance decomposition of lnY”	
	Forecast SE	Variance due to lnY” (%)	Forecast SE	Variance due to lnX” (%)
**1**	0.028948	0.000000	0.208065	51.24614
**3**	0.058611	2.521626	0.231910	47.72642
**5**	0.079387	4.145279	0.235713	49.06401
**7**	0.094325	5.009148	0.243856	52.20742
**10**	0.109780	5.662937	0.256814	56.29043

### 4.7 Granger-causality test

Finally, we conduct the Granger-causality test, which examines if the lagged value of one variable helps to predict other variables in the VAR model. The findings indicate that there is no evidence to support that the digital economy has a Granger-causality influence on the sports business (p = 0.3440), and similarly, the sports industry has no Granger-causality influence on the digital economy (p = 0.1869). Hence, it can be concluded that the rapid increase in the added value of the digital economy did not exert a definitive influence on driving the growth of the sports industry.

## 5. General discussion

In this study, we present evidence indicating that China’s sports industry exhibits promising signs of integration with the rapidly growing digital economy. In this respect, we apply the grey system theory, a research paradigm originating from physics, which is less often recognized but highly objective when it comes to handling limited data for decision making [75]. Previously, this method has been utilized to assess the integration of China’s digital economy, energy industry [76], forest industry [77], as well as economic growth [78]. It is useful to review and revisit the correctness of the methodological application. For instance, based on the grey relational degree of provincial integration, Yang and Xie showed that Guangdong province, Jiangsu province, Beijing, and Shanghai are in the forefront of the integration process of “internet + industry” in China [79]. Their finding aligns with other established economic or geographic-based methodologies [80, 81]. Hence, the utilization of gray relational analysis not only expands conventional research frameworks in assessing socioeconomic development but also provides cross validation in this regard. In this study, the sports sector demonstrates a higher level of integration in comparison to the other six key economic sectors. Thus, this study is the first of its kind that objectively shows a sectoral imbalance existing within the sports industry, which is one of the two added values of this study to the existing literature. Based on the oscillation of grey relational grades, we put up potential justifications for the present condition discussed in the preceding section.

Nevertheless, it is important to note that the observed correlation, whether high or low, should be interpreted solely as a statistical association and does not provide evidence of a causative relationship. In vast existing Chinese literature, however, it is often implied that the digital economy causes the growth of the sports industry. To demystify this aspect, a standard econometric method is employed in this study. From 2009 to 2021, no Granger-causality relationship has been observed between the sports industry and the digital economy. This suggests that despite the increasing popularity of online sales of sporting products, the expanding social influence of sports, and the growing awareness of exercise for healthspan, the level of digital transformation in the sports industry remains limited. Therefore, the available data indicate that the digital economy did not serve as the primary catalyst for the rapid growth of the sports industry between 2009 and 2021. This is the other added value to the existing literature, and more importantly, shows objective evidence for policy evaluation.

The next obvious question relates to the specific factor responsible for the 1033% surge in the total scale of the sports industry between 2009 and 2021 (see also S3 Table). As we recently discussed in depth [82], empirical data indicate that the impressive expansion of the sports industry can be attributed to two key drivers: the ongoing urbanization process in China and the strategic direction set by national policies, as outlined in Document No. 46 [21]. In brief, the process of urbanization in China, which was accelerated by the country’s accession to the WTO, has resulted in the upliftment of the vast rural population out of poverty in contemporary Chinese society. This improvement in their economic status has enhanced their capacity to engage in discretionary spending, leading to an exponential increase in sports consumption. Sports policies implemented after 2014 [83] further amplified this trend of sports consumption. The integration of the digital economy in this process was an add-on factor during this period, and its future bi-directional integration has the potential to act as an impetus for all-round and high-level industrial modernization towards 2035 [1], which could not be attainable by Web 2.0 and Industry 3.0 technologies.

Therefore, our systematic evaluation implies that China’s “sports + digital” complex is still in its early phase of development, presenting ample opportunities for future integration between the two. Meanwhile, this process will likely face many challenges, potentially leading to unforeseen consequences. We endeavor to adopt a strategic standpoint by retrospectively evaluating the past, grounding itself in the present, and envisioning the future, with the long-range objective of fostering the seamless integration of China’s sports industry and the digital economy. In response, we put forth the following policy recommendations.

Sectoral synergistic development: To address the sectoral imbalance, it is necessary to invest in core infrastructures across the sports industry. First, traditional sports sectors should collaborate with Chinese IT titans in the exploration of core technologies. Concurrently, governmental bodies at both national and local levels should implement supportive policies, such as tax exemptions and financial subsidies, for enterprises and research institutions engaged in the development of digital sports technologies. Second, it is necessary to rapidly employ existing digital technologies, especially big data and cloud computing. This will not only facilitate sports enterprises and professional sports clubs to engage in data-driven development, but also enhance the standards and efficacy of sports consumption. Third, the issue of sectoral imbalance may be amplified at the regional level. To share the digital economic development, it is necessary to harness the concepts obtained from the more advanced eastern regions for the benefit of the underdeveloped central and western regions in China.

Overtaking via esports IP: In the United States, the NBA has expanded its brand influence even more by establishing parallel sponsorship networks in the NBA 2K League [84]. This demonstrates an innovative digital business model for the Chinese sports industry. The dearth of premium professional sports events and IP in China severely constrains its sports industry from leveraging data for the creation of diverse and customized services. Consequently, this situation results in a negligible international influence of Chinese sports and its sports industry. We envision that traditional Chinese sports IPs are unlikely to experience strong international growth, while esports IP remains underdeveloped. Given this, there is a need to shift thinking to achieve an overtaking. Esports, being a modernized sports competition, has established a strong developmental framework and an extensive track record of tournaments in China. This has the true potential for the dissemination of China’s digital sports IP on the global stage. In addition, the massive amount of esports data has the potential to make a major difference in the marketing, product innovation, and data mining of esports IP, and in turn, a first-class young IP may facilitate the amplification of the Chinese sports industry’s global impact.

New economy and new regulation: The emergence of the “sports + digital” complex, as a new sector, business model, and economic form, is bound to have repercussions on the traditional sports legal framework and industry policies. At present, a unified, authoritative, and mandatory regulatory system has not been established yet. The discourse on the digital transformation of the sports industry is primarily found in policy documents such as the “Outline for Building a Leading Sports Nation,” which provides more guiding expressions rather than a legal framework. This will render numerous existing sports regulations inadequate in clarifying and addressing the intricacies of the sports industry within the digital realm, particularly regarding the definition of property rights and data ownership [85]. Given the predicament surrounding the inadequacy of the regulatory mechanism, it is critical to initiate efforts at the governmental level to expedite the establishment of a regulatory framework for the “sports + digital” complex.

## 6. Conclusions and outlook

This systematic evaluation answers two pressing issues for “building a strong sports nation” and “Digital China.” First, although the sports industry has emerged as a frontrunner in the integration of the digital economy within China’s key economic sectors, policymakers must address the evident sectoral imbalance. This finding calls for targeted policy actions to promote uniform digital integration across all sub-sectors of the sports industry. Subsequently, the VAR model addresses a common misconception that the growth of the sports industry was driven by the digital economy. The non-significant Granger-causality relationship indicates that the digital economy only had an added-on influence on the growth of the sports industry between 2009 and 2021. Hence, this finding can objectively challenge the academic consensus regarding the initial impact of digitization in the sports industry. The true significance of this finding lies in the fact that the current status highlights the immense potential for growth in the “sports + digital” complex, provided that certain shortcomings are addressed at the policy level. Collectively, these findings can aid the Chinese government and sports enterprises in pinpointing unique challenges and opportunities during the ongoing digital transformation of the sports industry.

Where does research go from here? We highlight two priority areas that we believe bear great potential for high-quality upgrade of this new economic engine. First, our grey relational analysis encompasses the macro and meso levels, but lacks a micro-level assessment of specific sectors, particularly the digitization of Chinese sporting manufacturing enterprises. The combined sales and manufacturing of sporting products contribute to almost 50% of the added value and total scale of the Chinese sports industry. China’s digital economy in 2022 amounted to 50.2 trillion yuan, or 41.5% of the country’s GDP, according to the National Bureau of Statistics of China. Presently, nearly all of the sales in this industry are generated in the domestic market [82]. In turn, there is significant potential for expansion by exploring new overseas markets. In light of China’s ongoing Digital Silk Road initiatives [86], Chinese sporting manufacturing enterprises should promptly transit their hybrid model to a full e-commerce model and actively engage in the overarching objective of establishing new manufacturing, retail, and service networks that connect Asia, Europe, and Africa. There is a pressing need for research that focuses on cultural and green consumerism [87, 88] and how they are being reshaped by digital technologies.

Second and to reiterate, this study solely focuses on the economic aspect of digital integration, the practical but narrow dimension of what counts as national competitiveness. The technological aspect of digitization is the fundamental basis for the sports industry and national innovation, hence arguably the most important factor in determining the process of “Digital China.” However, the evaluation of technological integration is more than plugging in conventional econometrics. There are any number of factors that determine how digitization a sector undergoes in this context, some of which are lagged economic factors (e.g., R&D expenses). Therefore, we suggest that future research assesses the following indicators associated with Industry 4.0 applications and Web 3.0 technologies as technological digitalization in the sports industry: R&D expenditures, subscription-based services or active user counts, and partnerships with esports, gaming industry, and digital health industry.

Last but not least, we leave two open questions for pioneers framing the next 100 years of a metaverse world: When everyone wants to be immersed, where is the exit for the sports industry? In turn, how may today’s meaningfulness of existence be improved?

## Supporting information

S1 Table Statistics of China’s digital economy, sports industry and other economic sectors between 2016 and 2021 (100 million CNY; 亿元). (DOCX)

S2 Table Statistics of China’s digital economy and sub-sectors within the sports industry between 2016 and 2021 (100 million CNY; 亿元). (DOCX)

S3 Table Statistics of China’s digital economy and the sports industry between 2009 and 2021 (100 million CNY; 亿元). (DOCX)
