# Supplementary material for: Systematic evaluation of integration between China’s digital economy and sports industry: Two-stage grey relational analysis and vector autoregressive model
Source: PLoS One. 2024 May 13;19(5):e0303572. doi: 10.1371/journal.pone.0303572 (PMC11090320; doi:10.1371/journal.pone.0303572)
Supplement: S1 Table — (DOCX) [file pone.0303572.s001.docx]

**Table S1. Statistics of China's digital economy, sports industry and other economic sectors between 2016 and 2021 (100 million CNY; 亿元).**

| **Indicator** | **2016** | **2017** | **2018** | **2019** | **2020** | **2021** |
| --- | --- | --- | --- | --- | --- | --- |
| Y. added value of the digital economy | 115575.5 | 133317.2 | 150576.7 | 170293.4 | 191447.3 | 213989.2 |
| X_1_. added value of the sports industry | 6475 | 7811 | 10078 | 11248 | 10735 | 12245 |
| X_2._ total scale of culture and related industries | 30785 | 34722 | 41171 | 44363 | 44945 | 52385 |
| X_3_. added value of tourism and related industries | 32979 | 37210 | 41478 | 44989 | 40628 | 45484 |
| X_4_. added value of the industrial sector | 245406.4 | 275119.3 | 301089.3 | 311858.3 | 312902.9 | 372575.3 |
| X_5_. added value of construction enterprises | 51498.9 | 57905.6 | 65493 | 70648.1 | 72444.7 | 80138.5 |
| X_6_. added value of wholesale and retail trades | 73724.5 | 81156.6 | 88903.7 | 95650.9 | 96086.1 | 110492.7 |
| X_7_. added value of hotels and catering services | 12306.1 | 13607.8 | 16520.6 | 17903.1 | 15285.4 | 17852.6 |
| X_8_. added value of financial intermediation | 57086 | 64623 | 70610.3 | 76250.6 | 83617.7 | 91205.6 |
