# Supplementary material for: Systematic evaluation of integration between China’s digital economy and sports industry: Two-stage grey relational analysis and vector autoregressive model
Source: PLoS One. 2024 May 13;19(5):e0303572. doi: 10.1371/journal.pone.0303572 (PMC11090320; doi:10.1371/journal.pone.0303572)
Supplement: S2 Table — (DOCX) [file pone.0303572.s002.docx]

**Table S2. Statistics of China's digital economy and sub-sectors within the sports industry between 2016 and 2021 (100 million CNY; 亿元).**

| **Indicator** | **2016** | **2017** | **2018** | **2019** | **2020** | **2021** |
| --- | --- | --- | --- | --- | --- | --- |
| Y’.added value of  the digital economy | 115575.5 | 133317.2 | 150576.7 | 170293.4 | 191447.3 | 213989.2 |
| X_1_’. added value of  sports management activities | 143.8 | 262.6 | 390 | 451.9 | 459 | 515 |
| X_2_’. added value of  sports competition performance activities | 65.5 | 91.2 | 103 | 122.3 | 103 | 129 |
| X_3_’. added value of  physical fitness and leisure activities | 172.9 | 254.9 | 477 | 831.9 | 736 | 892 |
| X_4_’. added value of  management of sports venues and facilities | 567.6 | 678.2 | 855 | 1012.2 | 808 | 1031 |
| X_5_’. added value of  sports brokerage and agency, advertising and exhibition, performance and design services | 17.8 | 24.6 | 106 | 117.8 | 98 | 119 |
| X_6_’. added value of  physical education and training | 230.6 | 266.5 | 1425 | 1524.9 | 1612 | 1795 |
| X_7_’. added value of  sports media and information service | 44.1 | 57.7 | 230 | 285.1 | 339 | 406 |
| X_8_’. added value of  sales, leasing and trade agency of sporting goods and related products | 2138.7 | 2615.8 | 2327 | 2562 | 2574 | 2955 |
| X_9_’. added value of  other sports services | 179.7 | 197.2 | 616 | 707 | 645 | 733 |
| X_10_’. added value of  manufacture of sporting goods and related products | 2863.9 | 3264.6 | 3399 | 3421 | 3144 | 3433 |
| X_11_’. added value of  construction of sports facilities | 50.3 | 97.8 | 150 | 211.9 | 217 | 236 |
