# Supplementary material for: Systematic evaluation of integration between China’s digital economy and sports industry: Two-stage grey relational analysis and vector autoregressive model
Source: PLoS One. 2024 May 13;19(5):e0303572. doi: 10.1371/journal.pone.0303572 (PMC11090320; doi:10.1371/journal.pone.0303572)
Supplement: S3 Table — (DOCX) [file pone.0303572.s003.docx]

**Table S3. Statistics of China's digital economy and the sports industry between 2009 and 2021 (100 million CNY; 亿元).**

| **Year** | **X’’. added value of the digital economy** | **Y’’. total scale of the sports industry** |
| --- | --- | --- |
| 2009 | 43251.6 | 2751.44 |
| 2010 | 54293.2 | 3206.47 |
| 2011 | 65676 | 3793.99 |
| 2012 | 74638.6 | 9526 |
| 2013 | 84940.8 | 11000 |
| 2014 | 95331.2 | 13574.71 |
| 2015 | 105383.5 | 17000 |
| 2016 | 115575.5 | 19000 |
| 2017 | 133317.2 | 22000 |
| 2018 | 150576.7 | 26579 |
| 2019 | 170293.4 | 29483 |
| 2020 | 191447.3 | 27372 |
| 2021 | 213989.2 | 31175 |
